# Supplementary material for: Chromothripsis during telomere crisis is independent of NHEJ, and consistent with a replicative origin
Source: Genome Res. 2019 May;29(5):737–49. doi: 10.1101/gr.240705.118 (PMC6499312; doi:10.1101/gr.240705.118)
Supplement: Supplemental Material [file supp_gr.240705.118_Supplemental_file_1.zip › contigs/annotated_contigs/DB38/contig.2.DB38_length_392_mean_cov_4.02806122449.docx]

**DB38_length_392_mean_cov_4.02806122449**

```
CCTATTTTCCAGATGAAGGAGCAGGCACAGTGAGGTTAATTAGCTTATTCAAGCTCACATGGCTACTTGAACTTAGATAATCTGTGTTC
 >chr3:159633816-159634067 - E=1e-139
ACTGGCACTTTAAACAACTATGCGTTCTTAGTGCTTTCTTTTCTTTTCTTTTGGTTTGTTTGTCTTAACCTTGATTTTATCATTTAACC

CCATATGGTGTATATCTGAGTTTGTGTTATATAGCATGACAGTGGGAATGTATACTGAGTTCTCCAAATGGGG|AACTCCT|AGGCTGG
                                                                                 >chr13
AGTGTGGTGGCATGATCTCAGCTCACTGCAACCTCTGCCTCCCAGGTTCAAGCGATTCTCCTGCCTCAGCCTCCCGAGTAGCTGGGATT
:26537372-26537506 - E=5e-39
ACAGGTGCCCACCACGACGCACAGTTAATCTTTGTATT
```
